# Altered tongue muscle contractile properties coincide with altered swallow function in the adult Ts65Dn mouse model of down syndrome

**DOI:** 10.3389/fneur.2024.1384572

**Published:** 2024-03-22

**Authors:** Tiffany J. Glass, John A. Russell, Erin H. Fisher, Marziyeh Ostadi, Nanyumuzi Aori, Y. Eugene Yu, Nadine P. Connor

**Affiliations:** ^1^Department of Surgery, Division of Otolaryngology, University of Wisconsin-Madison, Madison, WI, United States; ^2^Department of Communication Sciences and Disorders, University of Wisconsin-Madison, Madison, WI, United States; ^3^The Children’s Guild Foundation Down Syndrome Research Program, Department of Cancer Genetics and Genomics, Roswell Park Comprehensive Cancer Center, Buffalo, NY, United States; ^4^Genetics, Genomics and Bioinformatics Program, State University of New York at Buffalo, Buffalo, NY, United States

**Keywords:** Down syndrome, Ts65Dn, mouse, tongue, swallow, adult

## Abstract

**Purpose:**

Down syndrome (DS) is a developmental disability associated with difficulties in deglutition. The adult Ts65Dn mouse model of DS has been previously shown to have differences in measures of swallowing compared with euploid controls. However, the putative mechanisms of these differences in swallowing function are unclear. This study tested the hypothesis that the Ts65Dn genotype is associated with atypical measures of tongue muscle contractile properties, coinciding with atypical swallow function.

**Methods:**

Adult (5-month-old) Ts65Dn (*n* = 15 female, 14 male) and euploid sibling controls (*n* = 16 female, 14 male) were evaluated through videofluoroscopy swallow studies (VFSS) to quantify measures of swallowing performance including swallow rate and inter-swallow interval (ISI). After VFSS, retrusive tongue muscle contractile properties, including measures of muscle fatigue, were determined using bilateral hypoglossal nerve stimulation.

**Results:**

The Ts65Dn group had significantly slower swallow rates, significantly greater ISI times, significantly slower rates of tongue force development, and significantly greater levels of tongue muscle fatigue, with lower retrusive tongue forces than controls in fatigue conditions.

**Conclusion:**

Tongue muscle contractile properties are altered in adult Ts65Dn and coincide with altered swallow function.

## 1 Introduction

Dysphagia, or a disorder in swallowing, can be associated with critical problems in the safety and efficiency of eating and drinking that may lead to malnutrition, dehydration, aspiration pneumonia, and increased risk of death (1, 2). Risks for these problems increase with age. Adults with Down syndrome (DS) may experience dysphagia across oral, pharyngeal, and esophageal phases of swallowing (3) along with gastro-esophageal reflux disease, choking, and aspiration (2, 3). These issues can contribute to behavioral differences such as food refusal. Factors that may contribute to problems at the oral preparatory stage of the swallow in DS may include atypical tongue protrusion from the mouth during eating and drinking, inefficient mastication, and dental differences (4–7). Hypotonicity is often believed to be present and may be implicated in oromotor discoordination, inadequate lip sealing, and impaired tongue movement (8, 9). Pharyngeal phase swallowing problems include timing delays, which lead to a high risk of aspiration (4). In addition, uncoordinated patterns of breathing and swallowing can expose some individuals to higher risk of aspiration, presumably due to temporal incongruities that may result in presence of the bolus near an open airway (4). DS is also associated with primary or secondary esophageal motor disorders (10, 11). As adults with DS age, dysphagia might also be observed at earlier time points than in the general aging population (3). Accordingly, studies of putative mechanisms of dysphagia associated with adulthood and aging in DS are critical to development of effective management of care for individuals with DS experiencing signs of dysphagia.

Dysphagia in adults with DS may involve many underlying anatomical and functional factors including sensorimotor, orofacial, and developmental differences. Anatomically, craniofacial differences including an underdeveloped maxilla and shorter length and width of the palate in adults with DS may impact the safety and efficiency of feeding and swallowing (8, 12). People with DS may have differences in palatal morphology compared with people without DS, and it has been proposed that neuromotor dysfunction incurred by DS may result in less capacity to adjust tongue movement to accommodate these differences (8). For these and other reasons, studies of dysphagia in adults without DS cannot easily generalize to adults with DS. This population must be studied directly.

Mouse models of DS provide a means to study biomedical problems through experimental paradigms that are highly controlled for many variables, and that may involve mechanistic hypotheses and methodology that are not possible to perform in studies with human subjects. The Ts65Dn mouse model of DS displays a remarkable number of phenotypes applicable to characteristics of individuals with DS (13). The craniofacial phenotype of Ts65Dn has been extensively studied and has been reported to include relative reductions in size of the palate, as well as reduced size of the mandibles, which parallels aspects of craniofacial differences in many humans with DS (14). Applicable phenotypes also include traits indicative of muscle differences associated with DS, such as reduced grip strength, altered motor coordination, and reduced running and swimming speeds (15, 16). Genotype-specific differences have also been reported in a variety of measures of muscle biology (17–19). This suggests Ts65Dn is an appropriate model for studying DS-related craniofacial muscle impairments. However, the Ts65Dn mouse model does not entirely replicate the trisomy in humans with DS. The Ts65Dn partial trisomy is comprised of a portion of mouse chromosome 16 (Mmu16) and a section of chromosome 17, leading to a failure to model triplication of some of the genes found on Hsa21, in addition to including the triplication of other genes that are not applicable to DS (13, 20). Consequently, while this mouse model displays numerous phenotypes of interest, it may not encompass all of the genetic and molecular aspects of specific DS features. While work is on-going to compare phenotypes of Ts65Dn to phenotypes of newer mouse models that are anticipated to have superior molecular verisimilitude to DS, Ts65Dn is presently the only mouse model that has been both characterized for feeding and swallowing phenotypes, as well as having been found to demonstrate feeding and swallowing phenotypes that are translationally applicable to DS (21, 22). Ts65Dn exhibits differences from controls in body weight, chewing rates under some conditions, and some biological measures of specific muscles involved in swallowing and jaw movement (21, 23).

Although muscle weakness and hypotonia are typically assumed to be present in adults with DS and are often believed to impact muscles of the tongue, there are relatively few clinical studies and quantitative measures available for this area of study (9). Evaluation of pressure exerted by the tongue during swallowing in adults with DS has been studied through intra-oral pressure sensors (8). This has demonstrated that DS is associated with reductions in the duration and magnitude of tongue pressure on the median palate during swallowing, and that some of these reductions can be explained by differences of palate anatomy that are unique to DS. However, while some DS-specific tongue function phenotypes may occur as adaptations to anatomical differences of the palate, tongue function phenotypes may also be partly or wholly attributable to physiological, sensorimotor, or neuromotor differences of the tongue muscle system (8). Further, despite the fact that tongue strength is believed to be relevant to some aspects of swallowing ability under some circumstances, it is unclear if tongue muscle weakness coincides with significant swallowing phenotypes in DS. Murine models of DS provide access to a wide range of options for investigation of tongue muscle function that can circumnavigate some of these challenges, and which may provide some indication of whether differences in tongue muscle function coincide with differences in swallow function in DS.

The Videofluoroscopic Swallow Study (VFSS) is one of the gold standard diagnostic tests in humans for assessing oropharyngeal stages of swallowing. To adapt this method for mice and small rodents, a murine VFSS protocol was developed (24). This includes a reliable step-by-step test protocol for quantifying swallow metrics (24, 25). While VFSS has been used to identify significant differences in swallowing in the Ts65Dn model (21), VFSS alone gives incomplete information about muscle function during swallowing. Tongue muscle contractile properties can be directly evaluated in murine models as a function of neuromuscular electrical stimulation (NMES), either directly to muscle or with stimulation of the hypoglossal nerves (26). These methods allow quantification of twitch and tetanic forces, rate of force development, and half-decay times. These measures, indicative of tongue muscle force, temporal features of muscle contraction, and tongue muscle fatigue resistance can offer valuable insights into tongue muscle function. Thus, NMES and VFSS methods complement each other to evaluate structure and function of swallowing more precisely. The present study used VFSS and hypoglossal nerve stimulation in the adult Ts65Dn model of DS to test the hypothesis that the Ts65Dn genotype is associated with atypical measures of tongue muscle contractile properties, as well as atypical swallow function.

## 2 Methods

### 2.1 Mice

Mice for this study were generated from Ts65Dn breeding pairs comprised of one of the following: (1) Ts65Dn female (JAX 005252) and euploid F1 male (JAX 003647) purchased from the Jackson Laboratory, or (2) Ts65Dn females (JAX 005252) and euploid F1 males which were produced by mating C57BL/6JEiJ (JAX 000924) with C3Sn.BLiA-Pde6b+/Dn (JAX 003648). All breeding mice with the JAX strain numbers indicated were purchased directly from the Jackson Laboratory. Mice were maintained on the Harlan Teklad diet #7913, were genotyped for the presence or absence of the partial trisomy through Transnetyx^®^, or by our own team members using JAX Protocol 24762, and were genotyped for the Clcc1^<m1J>^ using JAX protocol 20574. Mice were analyzed at the age of 5 months (145–160 days of age). All procedures were conducted in accordance with an IACUC protocol approved through the University of Wisconsin, Madison, School of Medicine and Public Health Institutional Animal Care and Use Committee and the Roswell Park Comprehensive Cancer Center Institutional Animal Care and Use Committee.

### 2.2 Videofluoroscopy swallow studies

Mice were maintained on a reverse light cycle (27), and all behavioral experiments were performed between 9 am and 12 pm during the animals’ dark cycle.

#### 2.2.1 VFSS acclimation

Three days prior to the scheduled videofluoroscopy acquisition, mice were acclimated to Fritos™ mild cheddar cheese dip and Varibar^®^ Thin Honey Barium sulfate oral suspension 40% w/v barium. Days one and two of acclimation consisted of presenting the cheese to the mice in their home cages as they would encounter it during the videofluoroscopy session. Mice were allowed access to cheese until all cage mates had examined the cheese and attempted to feed, after which the cheese was removed from the cage. Day three of acclimation consisted of making a mixture of cheese and barium at a ratio of 2 mL cheese:1 mL barium suspension, which generated an extremely thick puree of IDDSI level 4. After mixing thoroughly, the cheese was presented as on days one and two. If mice exhibited signs of neophobia or disinterest on day one or two of acclimation, a food pellet was dipped into the cheese and left to remain in the cage overnight. The day prior to acquisition between 3:00 pm and 6:00 pm food was removed from the cages and mice were transferred to single housing. Food was withheld overnight for no more than 18 h, and *ad libitum* access to water was maintained.

#### 2.2.2 VFSS acquisition

A Genoray fluoroscopic x-ray system model ZEN-7000 was used to image mice in a lateral view. Images were captured in the Photon Fast Camera Viewer (PRV4) software with the following settings: frame rate 60fps, shutter speed 2.5 ms, resolution 1024×1024, Def 2 LUTS. On the C-ARM touchscreen the following settings were used: 55kVp, 3.0 mA, fluoroscopy mode, collimator 9-inch, hand and foot mode, dynamic noise reduction high mode. Once the settings were verified, footage comprised of a minimum of 7 s of continuous eating was acquired. Mice sometimes paused while eating, in which case acquisition was paused, and resumed when mice resumed eating. The field of view included the mouse’s entire head and, at minimum, upper body cavity with a view of the stomach, or the entire mouse body.

#### 2.2.3 VFSS analysis

Four parameters were evaluated in video acquisitions for each mouse: swallow rate (SR) which is defined as the number of swallows during two consecutive seconds of continuous, uninterrupted eating, inter-swallow interval (ISI) which is defined as the time between two successive, uninterrupted swallows (24), jaw excursion rate (JER), and jaw cycle-swallow ratio (JSR). All of these measures were quantified as in previous research (21, 24, 25). A minimum of three and up to five different instances of each measure were analyzed for each mouse, and then averaged to generate one data point for each mouse. JSR, or jaw cycle-swallow ratio is analogous to the lick-swallow ratio, and is defined as the number of jaw excursion cycles that occur during each ISI.

To verify appropriate inter-rater reliability of VFSS analysis, no fewer than 20% of VFSS videos in the study, arbitrarily selected and distributed across both sexes and both genotypes, were independently analyzed by two or more workers. Inter-class correlation coefficients (ICC) at or above 0.8, or Pearson correlation coefficients at or above 0.8 were confirmed for results from independent raters for all measures of analysis in this study.

### 2.3 Tongue muscle contraction studies

**Figure 1:**
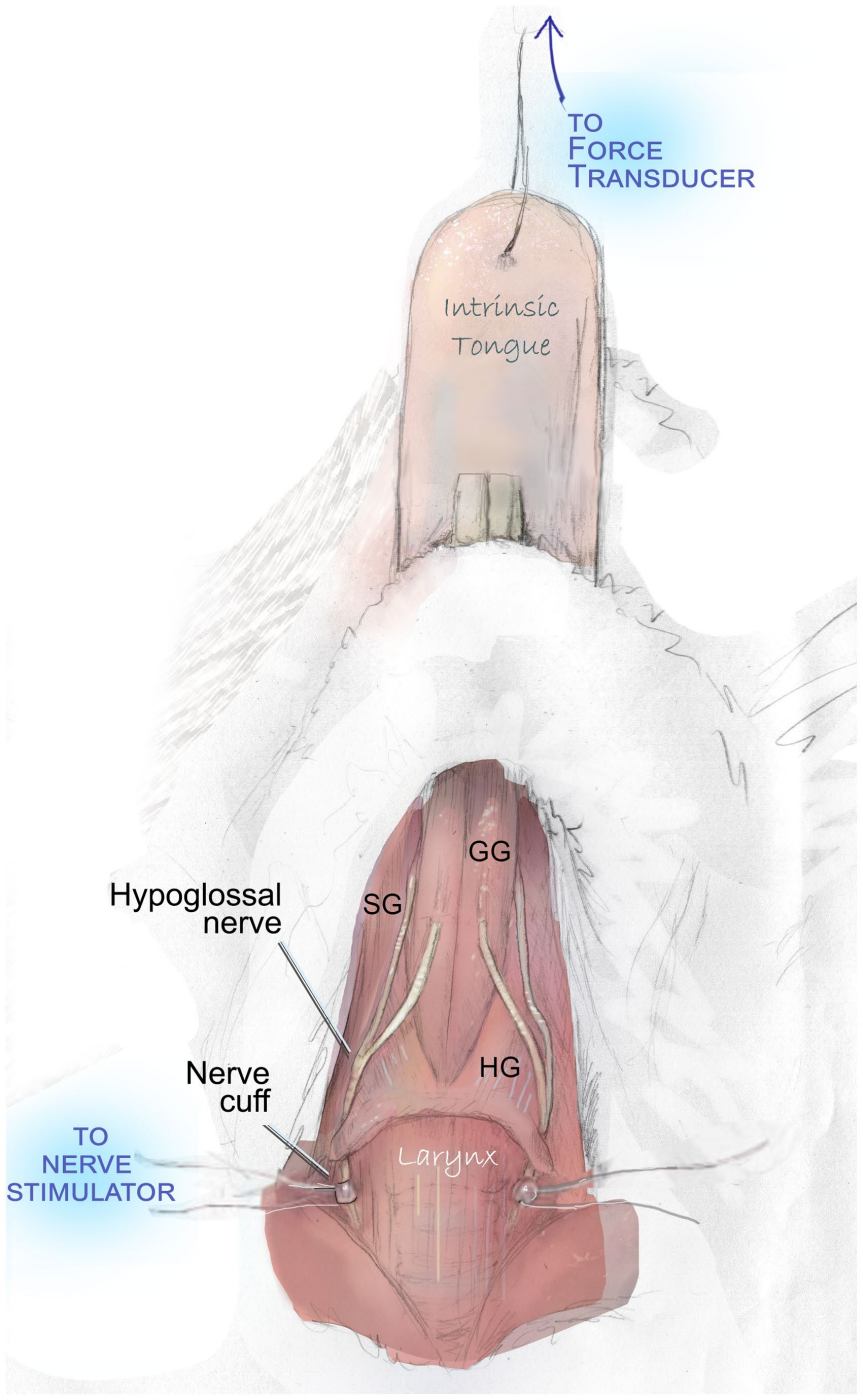
Hypoglossal nerve stimulation to evaluate tongue muscle contractile properties. With the exception of a midline incision and gentle retraction to permit access to the hypoglossal nerves, all overlying muscles and adjacent vessels are preserved intact during this procedure. They are omitted in this figure to demonstrate the course of the nerve and key tongue muscles. Bilateral stimulation of the hypoglossal nerves elicits contraction of the genioglossus (GG), hyoglossus (HG), styloglossus (SG), and intrinsic tongue muscles. This produces a net retrusive action. Contraction force (mN) is quantified with a force transducer.

After VFSS acquisition, mice were weighed and anesthetized with isoflurane and an I.P. injection 40–50 mg/kg sodium pentobarbital. Through close monitoring over the subsequent two-hour procedure, supplemental 5 mg/kg doses of sodium pentobarbital were administered intermittently to preserve a deep plane of anesthesia. Mice were placed in a supine position beneath an operating microscope. Following a midline incision of the skin over the location of the larynx and tongue base, salivary glands and the posterior digastric muscle were gently retracted, adjacent vessels were gently repositioned to preserve them, and a 5-mm section of the nerve was visualized. Custom-built silastic nerve cuffs were placed on the right and left hypoglossal nerves (Figure 1).

Following placement of hypoglossal nerve cuffs, cuff wires were connected to stimulus isolators through small alligator clips. A 10 mm needle and 6–0 or 7–0 silk suture were used to connect the tip of the tongue to an external force transducer to record elicited tongue muscle forces. Optimal line tension to yield maximum tongue muscle twitch forces was identified by stimulating the nerves with supramaximal stimulation (between 200 and 400 μA) and adjusting the length until the maximum force was obtained.

Measures of retrusive tongue properties were recorded during whole bilateral hypoglossal nerve stimulation using a data acquisition system (Aurora Scientific) as previously described (28), which generated the following experimental measures:

1. Maximum twitch force (mN) (the peak force generated from a single electrical stimulus), and the rate of force development (RFD) of twitch.
2. The relationship between muscle force and stimulation frequency (force-frequency curve) was demonstrated through stimulus frequencies of 1, 20, 40, 60, 80, 120, 160, 250, and 300 Hz. Maximum tetanic tension (mN) was the fused maximum tetanic force elicited from a range of stimulation frequencies.
3. Rate of force development from baseline to 50% of peak force (RFD1, Δ nM/s), rate of force development from 50% of peak force to maximum (RFD2, Δ nM/s), and rate of force loss (RFL, Δ nM/s) were normalized to maximum force values prior to analysis.
4. Data acquisition ended with a muscle fatigue protocol which consisted of a 1 s 80 Hz stimulus frequency (1:1 duty cycle) with force measurements collected at 0, 20, 40, 60, 80, 100, 120, 140, 160, 180, and 200 s, followed by a recovery period with data collection at 30, 60, 120, 300, 600, and 900 s. Results were calculated as the average of tetanic force (mN) relative to the initial tetanic tension (mN), expressed as a percentage of initial tension.

After completion of data collection, mice were euthanized through an injection of Euthasol.

### 2.4 Statistics

A target sample size of 14 mice per group was anticipated to provide sufficient power to reveal biologically meaningful differences in measurement variables based on effect sizes of the ISI and JSR measures in a prior study of swallow function in Ts65Dn (21). However, group sizes for some measures in some groups varied slightly due to experimental factors such as incidental attrition or sporadic technical artifact. Data were analyzed by 2-way ANOVA to interrogate main effects for the Ts65Dn genotype and sex, as well as interaction effects. Data that failed to conform to the assumptions of ANOVA were log transformed prior to analysis to comply with requirements for heteroscedasticity and normality. Because JAX recommends evaluating Clcc1^<m1J>^ genotype in Ts65Dn, exploratory analysis of the impact of the Clcc1^<m1J>^ genotype on the ISI measure of swallow function was performed through 2-way ANOVA in data pooled from male and female mice. Significance was set at α = 0.05.

## 3 Results

### 3.1 Videofluoroscopy swallow studies

**Figure 2:**
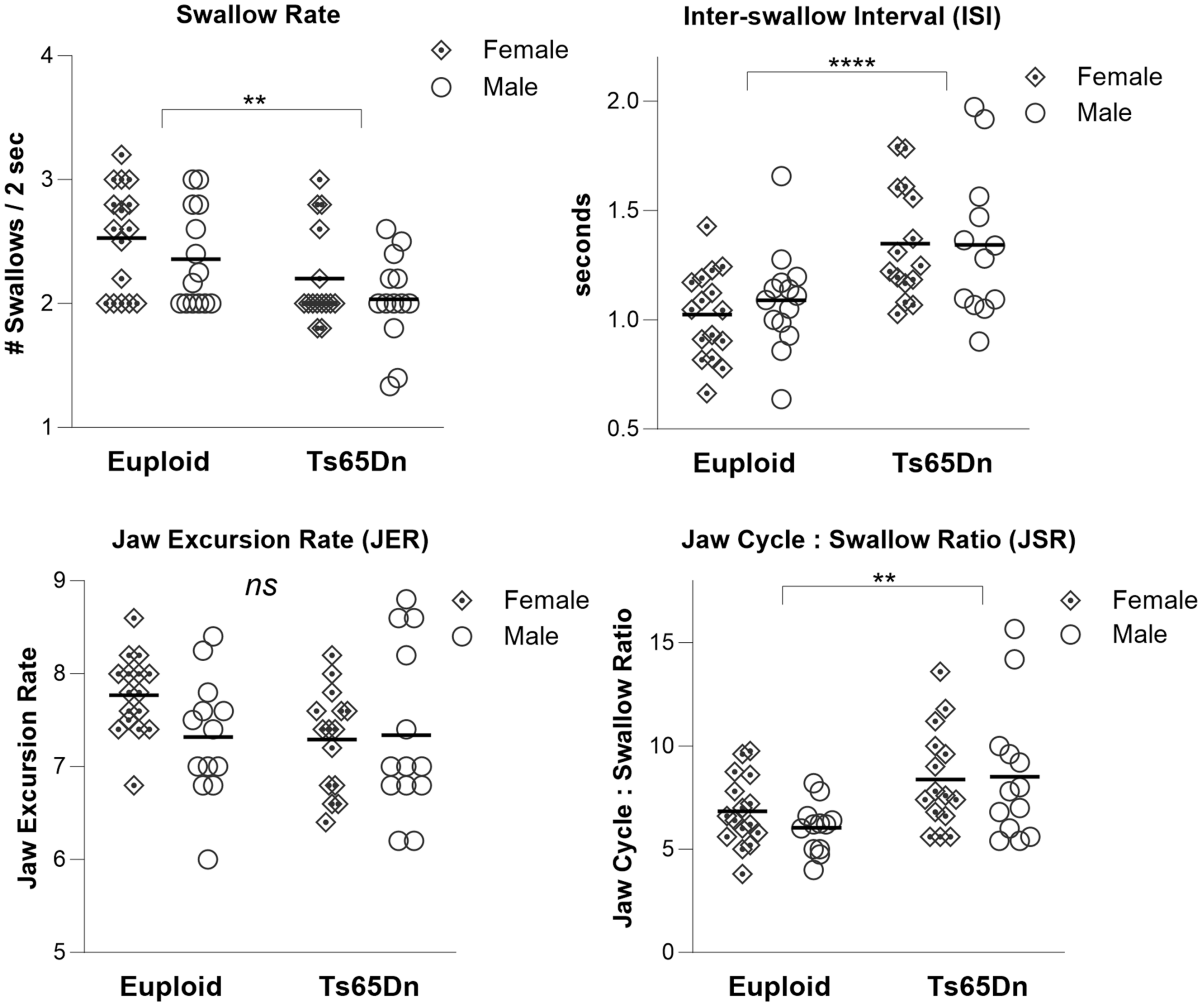
Swallow phenotypes in Ts65Dn at 5 months of age. Ts65Dn have significantly reduced swallow rates compared to euploid control. Ts65Dn have significantly increased inter-swallow intervals compared to euploid control. Ts65Dn have significantly greater number of jaw cycles preceding each swallow compared to euploid control. No significant differences between groups were detected in jaw excursion rates. *N* = 12–16 mice per group. ** = *p* ≤ 0.01, *** = *p* ≤ 0.001, **** = *p* ≤ 0.0001.

During continuous eating of a puree texture, Ts65Dn showed significantly slower swallow rate than euploid (*F*(1,54) = 9.79, *p* = 0.003). Ts65Dn also showed significantly longer inter-swallow intervals, or the amount of time elapsing between consecutive swallows (*F*(1, 53) = 17.71, *p* < 0.0001). Ts65Dn showed significantly larger JSR values, indicating a greater number of jaw cycles per swallow (*F*(1,52) = 11.25, *p* = 0.0015). However, there were not significant genotype-specific differences in the jaw excursion rate (JER), which reflects the rate of jaw movements during eating (Figure 2). For all VFSS measures, there were no significant effects for sex, and no significant interactions between genotype and sex. Exploratory analysis suggested ISI measures in Ts65Dn were not significantly impacted by Clcc1^<m1J>^ genotype (Supplementary Figure S1).

### 3.2 Tongue muscle contraction studies

**Figure 3:**
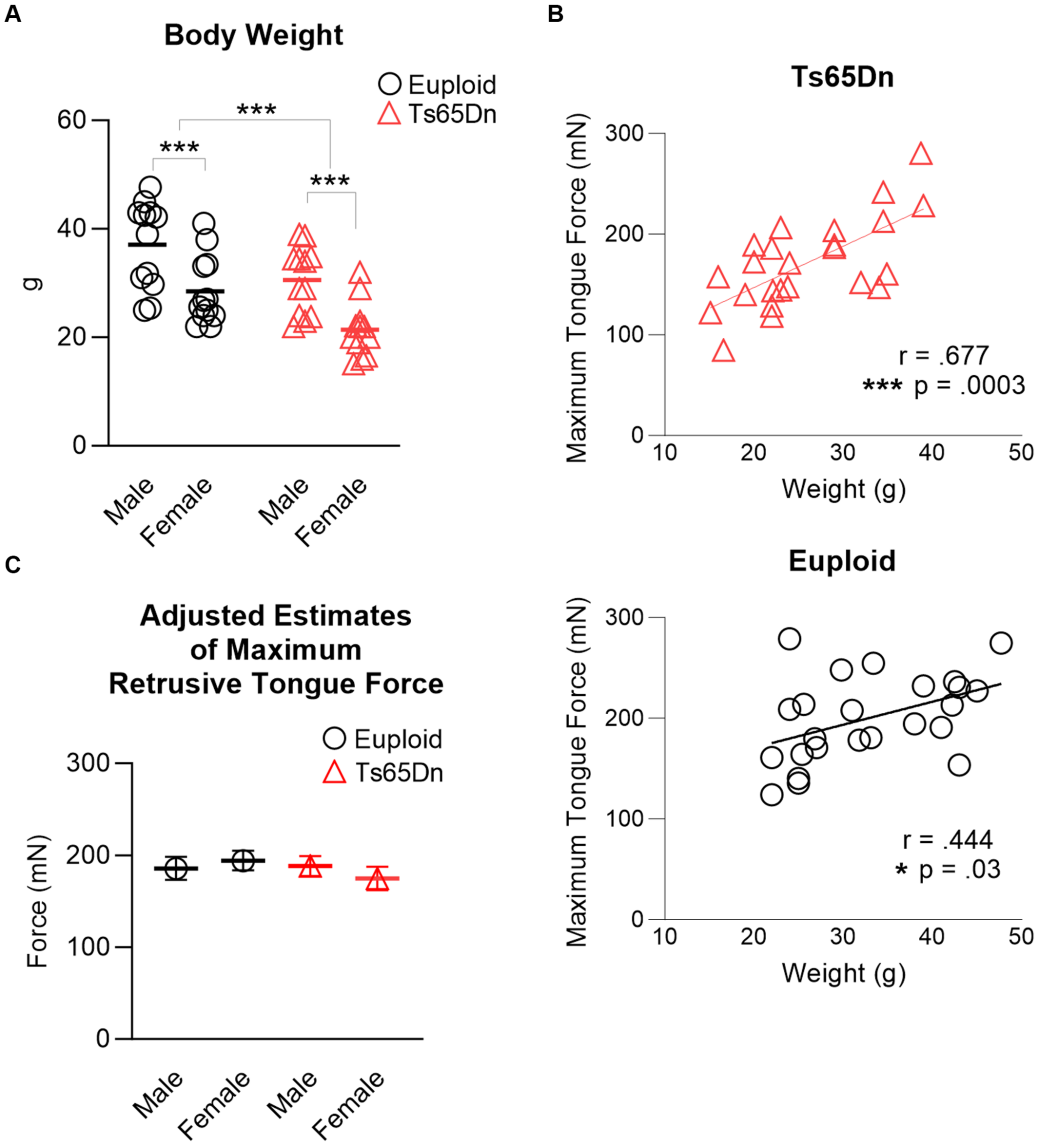
Maximum retrusive tongue muscle force. **(A)** Animal sizes were significantly different between groups. Each data point indicates one mouse. **(B)** Significant moderate correlations between animal size and maximum retrusive tongue force suggest that force differences between Ts65Dn and euploid are attributable to animal size. Each data point indicates one mouse. **(C)** Adjusted estimates of maximum retrusive tongue forces resulting from analysis covarying by animal weight were not significantly different between groups. Each data point indicates the group mean. Standard error is shown. *N* = 12 mice per group. *** = *p* ≤ 0.001.

There were significant differences in body weight associated with both sex and genotype, in the absence of interaction between genotype and sex. Ts65Dn weighed significantly less than euploid (F (1,44) = 13.37, *p* < 0.001) and females weighed significantly less than males (F (1,44) = 22.547, *p* < 0.001) (Figure 3A). Analysis through ANOVA suggested that Ts65Dn had significantly lower maximum forces than euploid (F (1,44) = 5.772, *p* = 0.021) and females had significantly lower maximum forces than males (F (1,44) = 5.847, *p* = 0.020). However, there was a moderate and significant relationship between body weight and maximum retrusive tongue force in both euploid (*p* = 0.03, r = 0.444) and Ts65Dn (*p* = 0.0003, r = 0.677) (Figure 3B). Therefore, it is likely that these significant differences in tongue force are attributable to overall animal size, and by extension, overall muscle size, rather than to physiological differences between groups in these comparisons. To accommodate the possibility that differences in animal size could confound group comparisons of tongue force measures, maximum retrusive tongue force was analyzed with body weight as a covariate. Analysis with a covariate of weight indicated no significant differences in maximum retrusive tongue force due to genotype or sex, and an absence of significant interactions between genotype and sex (Figure 3C). Similarly, there was a moderate and significant relationship between body weight and maximum twitch force in both Ts65Dn (*p* = 0.022, r = 0.464) and euploid (*p* = 0.013, r = 0.500). However, analysis with a co-variate of weight indicated no significant differences in maximum twitch force due to genotype or sex, and an absence of significant interactions between genotype and sex.

**Figure 4:**
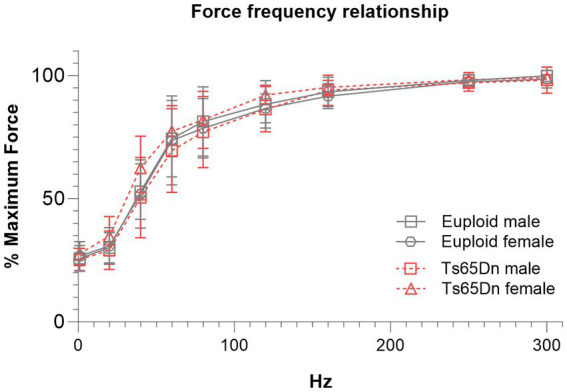
Ts65Dn show no significant differences in force frequency measures. Each data point indicates the group mean. Error bars indicate SD. *N* = 11–12 mice per group.

There were no significant differences due to genotype or sex in measures of the force-frequency relationship when forces were normalized to each individual’s maximum retrusive tongue force (Figure 4).

**Figure 5:**
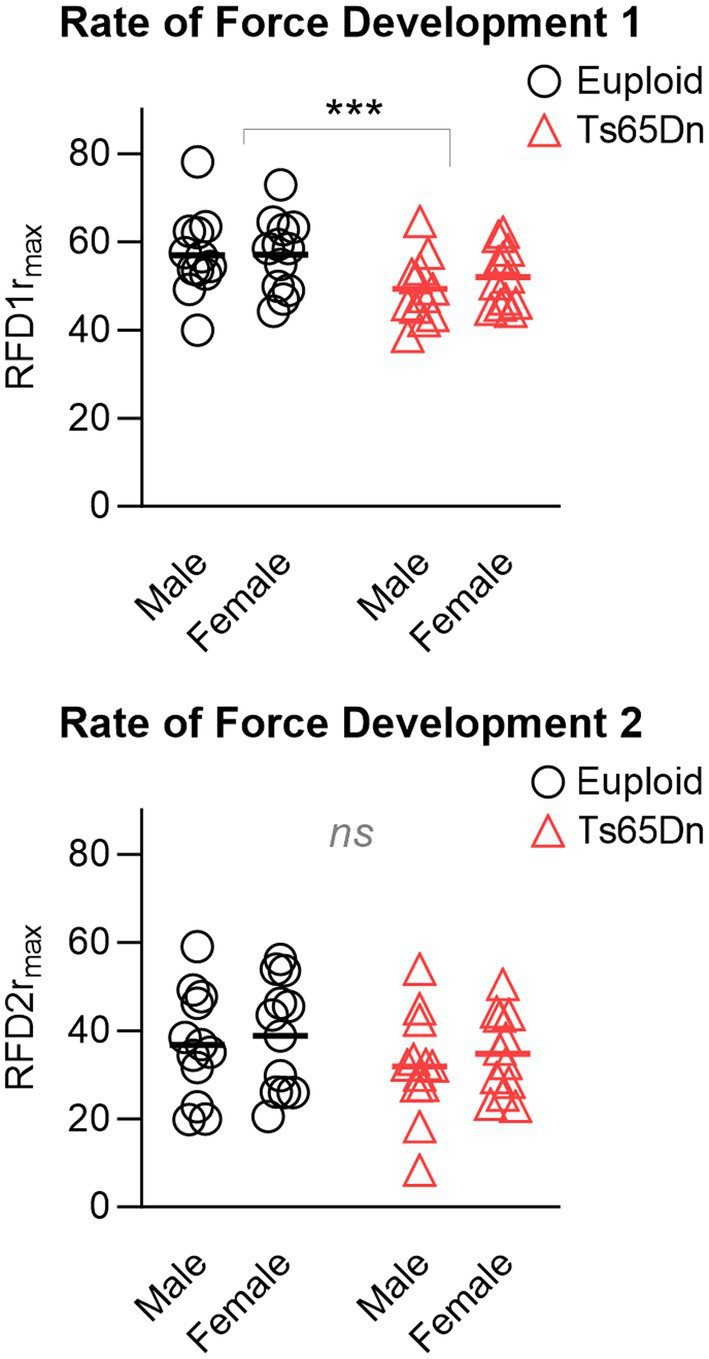
Ts65Dn show significantly lower values for RFD1 than euploid control, however, there are no significant genotype-specific differences in RFD2. RFD = Rate of force development. r_max_ indicates relative to maximum force. *N* = 12 mice per group. Each data point indicates one mouse. Bars indicate the group mean. *** = *p* ≤ 0.001.

In the measures of the rates of force development for tetanus normalized to the maximum force, Ts65Dn showed significantly lower values for RFD1 than euploid control (F (1,44)=7.709, *p* = 0.008), in the absence of differences due to sex or significant interactions between sex and genotype. However, there were no significant differences between groups in RFD2 (Figure 5), and there were no significant differences due to genotype or sex in RFL measures. Ts65Dn also showed significantly lower values than euploid for the RFD of twitch (F (1,44)=6.915, *p* = 0.012). Females showed lower values than males for the RFD of twitch (F (1,44)=5.749, *p* = 0.021), in the absence of significant interactions between genotype and sex.

**Figure 6:**
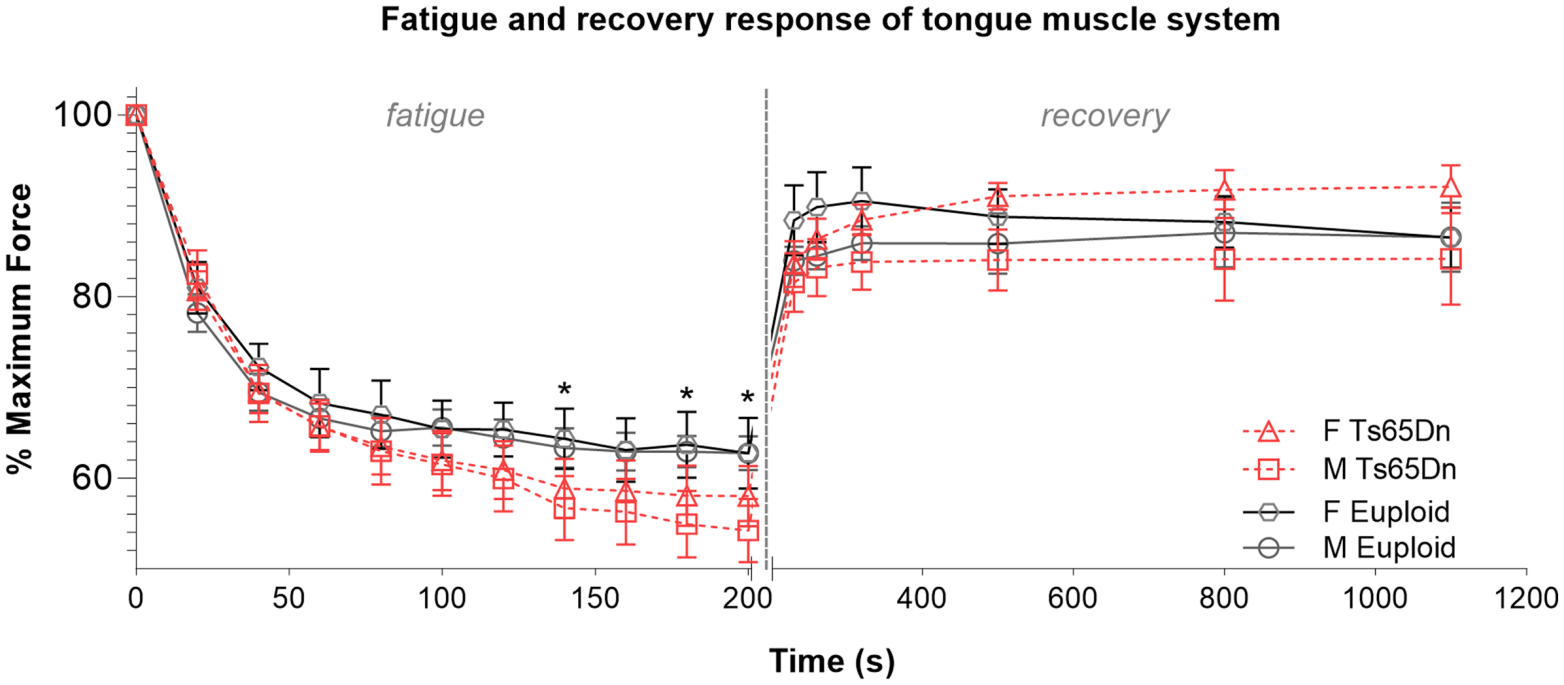
Ts65Dn show significantly increased retrusive tongue muscle fatiguability. *N* = 11–13 mice per group. Each data point indicates the group mean. Error bars indicate SEM. * = *p* < 0.05.

In evaluation of muscle fatigue elicited by repeated hypoglossal nerve stimulation, Ts65Dn showed significantly lower levels of retrusive tongue force than euploid after 140 s of stimulation (F (1,44) = 4.473, *p* = 0.040), 180 s of stimulation (F (1,44) = 4.283, *p* = 0.044), and 200 s of stimulation (F (1,44) = 4.133, *p* = 0.048), which suggests that onset of tongue muscle fatigue occurs more quickly with hypoglossal nerve stimulation in Ts65Dn than euploid (Figure 6). In analysis of muscle fatigue there were no significant differences due to sex, and no significant interactions between genotype and sex. There were also no significant differences between groups at timepoints during the recovery phase.

## 4 Discussion

The goal of this work was to test the hypothesis that the Ts65Dn genotype is associated with atypical measures of swallow function and tongue muscle contractile properties. Our findings were compatible with this hypothesis. We found that during continuous eating of a puree texture, adult Ts65Dn demonstrated significantly slower swallow rates and significantly longer time intervals between consecutive swallows. In addition, Ts65Dn demonstrated slower rates of tongue force development and a more rapid onset of tongue muscle fatigue during evoked retrusive actions. While Ts65Dn had lower values of retrusive tongue forces than euploid control, there was also a significant relationship between animal size and tongue force in both Ts65Dn and control groups, such that presumably smaller muscles generated commensurately lower forces. This is an important factor to consider because DS is often associated with slower growth and thus smaller body size than euploid. Also, sex-specific differences in body size occur in DS as well as in those without DS (29–31). When tongue forces were normalized to body size for analysis, such that tongue forces were analyzed in the context of body weight, no significant differences in tongue force were observed between Ts65Dn and control. Collectively, these findings suggest that while tongue forces are appropriate to animal size in Ts65Dn, Ts65Dn have significant increases in fatiguability of tongue muscles and are slower to develop force in tongue muscles. While it is plausible that these muscle contraction phenotypes may be implicated in increased ISI and decreased swallow rates in Ts65Dn, future studies are required to determine whether there is a causal relationship between these tongue muscle contractile phenotypes and swallowing phenotypes in Ts65Dn.

Properties of swallowing characterized in this study independently replicated previous findings of the adult Ts65Dn swallowing phenotype (24). While the causes of this phenotype remain unknown, slower swallow rates do not coincide with significantly slower jaw movements during food procurement, as evaluated through the JER measure. Compared to controls, Ts65Dn had significantly more jaw excursion cycles prior to each swallow as demonstrated in the JSR measures. A possible explanation is that despite maintaining typical jaw cycle rates, Ts65Dn may have reduced efficiency of oral processing for collection and transfer the bolus prior to initiation of the swallow. Oral processing depends upon sensorimotor coordination of the tongue muscle system. To examine the possibility that function of the tongue muscle system is altered in Ts65Dn, a hypoglossal nerve stimulation paradigm was used to quantify muscle contractile properties during experimentally elicited tongue retrusion. While there was no evidence of genotype-specific weakness in the tongue muscle system upon initial stimulation, Ts65Dn showed significant reductions in the rates of retrusive tongue muscle force development. In addition to having slower rates of force development, Ts65Dn also showed significant increases in fatiguability of tongue retrusion upon repeated stimulation, manifested as significant reductions in the ability to maintain retrusive tongue muscle force upon repeated muscle contraction demands. Heightened susceptibility to muscle fatigue in the tongue may be particularly concerning for deglutition efficacy in Ts65Dn because the swallowing phenotype in this model entails significantly greater oral processing times prior to each swallow than for controls. In some older human populations the consumption of even a typical meal may cause subsequent delays in swallowing initiation, possibly due to fatigue incurred by consuming the meal (32). However, it is worth noting that tongue fatigue in our experimental system did not occur immediately upon stimulation, but rather, occurred only after stimulation for over 2 minutes, which may not have been entirely applicable to the duration of tongue activity elicited during VFSS sessions which typically lasted only several seconds. Therefore, one may speculate that findings of precocious tongue fatigue in Ts65Dn may be more applicable to more naturalistic deglutition settings, such as in *ad libitum* feeding occurring in standard husbandry conditions. In those settings, more naturalistic eating bouts may unfold over much long periods of time. It is also reasonable to consider that longer oral processing times in Ts65Dn may exacerbate tongue muscle fatigue during eating. In humans, it has been reported that oral processing demands of consuming meals may elicit fatigue-induced reductions in tongue strength in some populations (33, 34). From the standpoint of translational DS research, future work to better understand the causes and the consequences of tongue muscle fatigue in DS may support improved understanding of deglutition phenotypes in this syndrome.

In both humans and murine models, tongue protrusion and potentially co-activation of tongue muscles are involved in successful deglutition (35–37). The puree food used in this study was acquired by mice through licking, which requires at least protrusion of the tongue from the oral cavity to procure the food, retrusion of the tongue into the oral cavity, and collection of a cohesive food bolus followed by a number of key muscle actions that alter pressures within the upper aerodigestive tract for posterior transport of the bolus leading the swallow. Tongue protrusion is accomplished through the genioglossus (an extrinsic tongue muscle), and may be aided through activity of the transverse and verticalis (intrinsic tongue muscles), which are thought to promote elongation of the intrinsic tongue (36). Conversely, tongue retrusion is accomplished through the styloglossus and hyoglossus (extrinsic tongue muscles), and contraction of the superior longitudinal and inferior longitudinal (intrinsic tongue muscles) that may act to shorten the intrinsic tongue when contracted (37, 38). Because of the complex interdigitation of these tongue muscles and the fact that they work synergistically during tongue movement and shape changes of the intrinsic tongue, it is not uncommon for physiological studies of the tongue to interrogate the tongue muscle system intact. This strategy was used in the present study, in which bilateral stimulation of the hypoglossal nerves in the intact tongue muscle system elicited contraction of both protrusive and retrusive tongue muscles, ultimately resulting in net retrusive forces, which were then quantified. The benefit of this approach is it generates information about the physiological properties of the intact muscle system in the mice. The substantial limitation of this approach is that it is unable to clearly identify which components of the tongue muscle system may be primarily responsible for contractile phenotypes of interest. Therefore, the precise muscular etiologies of the genotype-specific differences in muscle fatigue in tongue retrusion identified in this study remain somewhat unclear, as described below.

It is possible that susceptibility to muscle fatigue in Ts65Dn may be related to differences of basic muscle biology associated with this syndrome, including phenotypes related to constitutive oxidative stress, energy metabolism, and muscle bioenergetics, as characterized previously (19, 39, 40). A prior study of the soleus muscle in Ts65Dn identified typical contraction forces in Ts65Dn soleus without fatigue, but found reduced muscle forces during recovery from fatigue (19). Since both a prior study of limb muscle as well as the current study of tongue muscle found no evidence of muscle weakness under typical conditions, but did find genotype-specific reductions in force related to fatigue conditions, it can be speculated that Ts65Dn may have global muscle phenotypes specific to fatigue.

Because the present study measured net retrusive forces that occurred as a result of co-activation of tongue protrusors and retrusors, an alternative possibility to consider is that precocious fatigue in tongue retrusion in this context could also result from an imbalance in the contribution of tongue protrusor muscles relative to retrusor muscles, or a larger proportion of force generated by tongue protrusors in Ts65Dn relative to controls. There are presently few, if any, studies elucidating the relative contributions of tongue protrusor muscles and tongue retrusor muscles to the anatomy and function of the tongue muscle system in DS. However, there is extensive information in the literature that DS may entail atypical co-activation of agonist and antagonistic muscles, and it is possible to speculate that this may occur in the tongue muscle system (9). In conjunction with further studies of tongue physiology, future work may consider avenues through which such an imbalance between tongue protrusors and tongue retrusors could occur in DS. One example of an avenue for future investigation may be the somatotopic organization of the hypoglossal motor nucleus, the anatomical region in which motor neurons in the brainstem responsible for the control of intrinsic and extrinsic tongue muscles of tongue protrusion and tongue retrusion. This somatotopic organization is directed in part through expression of the transcription factor *Runx1*, which is located on the 21st human chromosome and has been reported to have atypical expression levels in DS (39). *Runx1* has been reported to promote expansion of hypoglossal motor neurons controlling tongue protrusor muscles specifically (41). Several studies have characterized a preponderance of tongue protrusion phenotypes in DS, or tendencies to protrude the tongue in situations that would not typically elicit tongue protrusion in individuals without DS (7, 42, 43). Historically, it has been widely recognized that many differences in tongue movement and positioning in DS may be at least partly attributable to craniofacial etiologies, including differences in palate morphology and a disproportionately smaller oral cavity, which could impose substantial anatomical constraints on tongue movement (8, 44). However, the present study evaluated retrusion of the tongue muscle system through a nerve stimulation paradigm in which the tongue was not constrained within the oral cavity, and in which there were neither behavioral nor volitional influences on tongue movement. Therefore, it is possible that the retrusive phenotype reported here could be attributable to differences of neuromuscular anatomy or physiology of the tongue in Ts65Dn.

Using information gained from basic studies of this area to ultimately improve outcomes for individuals with DS will require engagement with a variety of challenges. The clinical study of tongue muscle function and swallowing disorders in adults with DS has some logistical limitations. These include limited geographical availability and capacities of specialized multidisciplinary clinical care centers for DS, which creates barriers for some adults with DS to receive health care services aligned with best practices (45, 46), accessibility barriers that disproportionately disenfranchise individuals with communication support needs who may require special accommodation in order to communicate with medical providers (47), which may be applicable to many individuals with DS (44, 48, 49), and risks for disconnects between researcher expectations and the lived experiences of some disabled study participants (50). In light of these systemic challenges, the continued use of animal models of DS for basic discovery of underlying mechanisms of dysphagia is one of many strategies that can be used to ultimately advance more equitable inclusion of people with DS in the benefits of basic dysphagia research.

## Data availability statement

The original contributions presented in the study are included in the article/Supplementary material, further inquiries can be directed to the corresponding author.

## Ethics statement

The animal study was approved by University of Wisconsin, Madison, School of Medicine and Public Health Institutional Animal Care and Use Committee and the Roswell Park Comprehensive Cancer Center Institutional Animal Care and Use Committee. The study was conducted in accordance with the local legislation and institutional requirements.

## Author contributions

TG: Conceptualization, Data curation, Formal analysis, Funding acquisition, Investigation, Methodology, Project administration, Resources, Supervision, Validation, Visualization, Writing – original draft, Writing – review & editing. JR: Conceptualization, Data curation, Formal analysis, Investigation, Methodology, Validation, Writing – original draft, Writing – review & editing. EF: Data curation, Investigation, Methodology, Project administration, Supervision, Writing – original draft, Writing – review & editing. MO: Validation, Writing – original draft, Writing – review & editing. NA: Investigation, Writing – original draft, Writing – review & editing. YY: Conceptualization, Funding acquisition, Methodology, Project administration, Resources, Writing – original draft, Writing – review & editing. NC: Conceptualization, Funding acquisition, Project administration, Resources, Supervision, Writing – original draft, Writing – review & editing.
