# Supplementary material for: Altered tongue muscle contractile properties coincide with altered swallow function in the adult Ts65Dn mouse model of down syndrome
Source: Front Neurol. 2024 Mar 22;15:1384572. doi: 10.3389/fneur.2024.1384572 (PMC10995394; doi:10.3389/fneur.2024.1384572)
Supplement: Supplementary file 1 [file Table_1.DOCX]

## Supplementary Figure

**
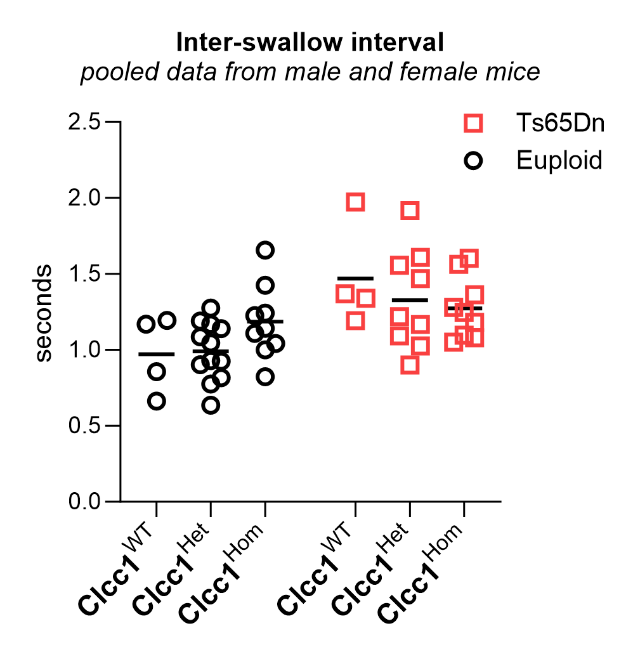
**

**Supplementary Figure 1.** **Inter-swallow Interval (ISI) (seconds) is shown for each genotype subtype of the** ***Clcc1^m1J^* mutation. Data from male and female mice were pooled for exploratory analyses due to the relatively low sample sizes resulting from division of data into six genotype groups. 2-way ANOVA to evaluate the impact of *Clcc1^m1J^* mutation and Ts65Dn genotype on ISI failed to detect a significant impact of the *Clcc1^m1J^* mutation on ISI. Results confirm a significant impact of the Ts65Dn genotype on ISI (p=.0005), in the absence of a significant interaction effect between *Clcc1^m1J^* mutation status and Ts65Dn genotype. Each data point indicates results for one mouse. WT = wild type, Het = heterozygous, Hom = homozygous.**
